# Quantifying similarity between motifs

**DOI:** 10.1186/gb-2007-8-2-r24

**Published:** 2007-02-26

**Authors:** Shobhit Gupta, John A Stamatoyannopoulos, Timothy L Bailey, William Stafford Noble

**Affiliations:** 1 Department of Genome Sciences, University of Washington, 1705 NE Pacific Street, Box 355065, Seattle, WA 98195, USA; 2 Institute for Molecular Bioscience, University of Queensland, Brisbane, QLD 4072, Australia; 3 Department of Computer Science and Engineering, University of Washington, 185 Stevens Way, Box 352350, Seattle, WA 98105, USA

## Abstract

A common question within the context of *de novo *motif discovery is whether a newly discovered, putative motif resembles any previously discovered motif in an existing database. To answer this question, we define a statistical measure of motif-motif similarity, and we describe an algorithm, called Tomtom, for searching a database of motifs with a given query motif. Experimental simulations demonstrate the accuracy of Tomtom's *E *values and its effectiveness in finding similar motifs.

## Abstract 2

Tomtom allows for the statistical measurement of similarity between pairs of motifs, thereby enabling searching a motif database with a motif query.

## Background

Discovering and characterizing DNA and protein sequence motifs are fundamental problems in computational biology. Here, we use the term 'motif' to refer to a position-specific probability matrix that describes a short sequence of amino acids or nucleotides that is important to the functioning of the cell. For example, the regulation of transcription requires sequence-specific binding of transcription factors to certain *cis*-acting motifs, which typically are located upstream of transcriptional start sites [1]. On the other hand, protein sequence motifs might correspond to active sites in enzymes or to binding sites in receptors [2].

**Figure 1:**
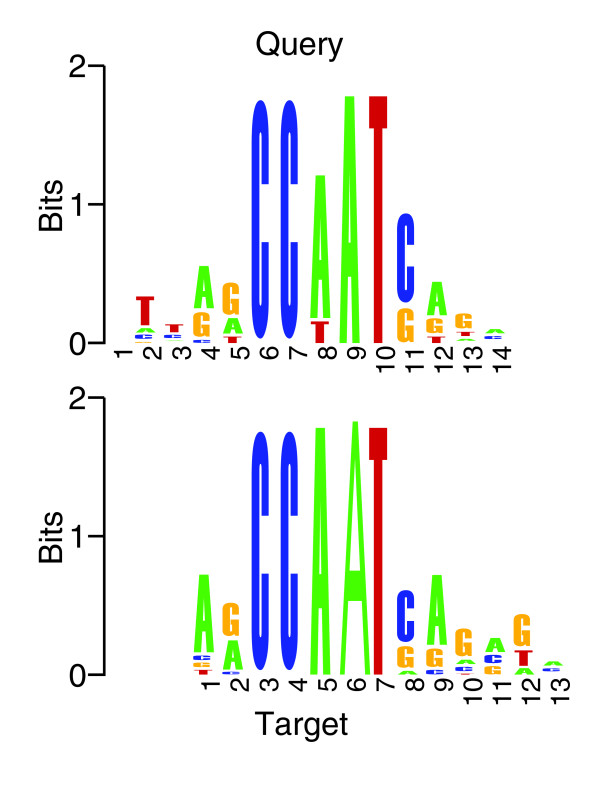
An aligned pair of similar motifs. The query and target motifs are both derived from JASPAR motif NF-Y, following the simulation protocol described in the text. Tomtom assigns an *E *value of 3.81 × e^-10 ^to this particular match. The figure was created using a version of seqlogo [26], modified to display aligned pairs of Logos.

A wide variety of statistical methods have been developed to identify sequence motifs in an unsupervised manner from collections of functionally related sequences [3]. In addition, databases such as JASPAR [4], TRANSFAC [5], and BLOCKS [6] can be used to scan a sequence of interest for known DNA or protein motifs. In this work we develop a statistical method for comparing two DNA or protein motifs with one another. This type of comparison is valuable within the context of motif discovery. For example, imagine that you are given a collection of promoter regions from genes that share similar mRNA expression profiles, and that a motif discovery algorithm identifies a motif within those promoters. Often, the first question you would ask is whether this new motif resembles some previously identified transcription factor binding site motif. To address this question, you need a computer program that will scan a motif database for matches to your new (query) motif. The program must consider all possible relative offsets between the two motifs, and for DNA motifs it must consider reverse complement matches as well. An example alignment between two similar motifs is shown in Figure 1. An alternate use for a motif comparison program would be to identify and then eliminate or merge highly redundant motifs within an existing motif database.

We are not the first to describe a method for quantifying the similarities between pairs of motifs. Pietrokovski [7] compared protein motifs using a straightforward algorithm based on the Pearson correlation coefficient (PCC). Subsequently, Hughes and coworkers [8] applied a similar method to DNA motifs. Wang and Stormo [9] introduced an alternate motif column comparison function, termed the average log-likelihood ratio (ALLR). More recently, Schones and coworkers [10] introduced two motif similarity functions, one based on the Pearson *χ*^2 ^test and the other on the Fisher-Irwin exact test (FIET). They showed that these two new functions have better discriminative power than the PCC and ALLR similarity functions. In addition, multiple research groups have used Kullback-Leibler divergence (KLD) to compare motifs [11-13], and Choi and coworkers [14] used euclidean distance (ED) to compare protein profiles. Finally, Sandelin and Wasserman [15] used their own column comparison function (SW) within the context of a dynamic programming alignment approach to compare DNA motifs. This method differs significantly from all other DNA-motif based approaches in the sense that it allows gaps in the motif-motif alignments.

In this report we focus on ungapped alignments of motifs. We describe a general method for accurately modeling the empirical null distribution of scores from an arbitrary, additive column comparison function. We estimate the null distribution of scores for each column in a 'query' motif using the observed scores of aligning it with each motif column in a database of 'target' motifs. Using a dynamic programming algorithm inspired by earlier work on searching a sequence database with a motif [16-18], we estimate the null distribution of the sum of scores for any range of contiguous columns in the query motif. This makes it possible for the user to determine whether the motif comparison score between the query motif and a particular target motif is statistically significant. Previous methods begin by defining a score between two motif columns, and then they combine these scores either by summing (as we do) [7-9,14] or by taking the mean [11-13] or geometric mean [10] of the column scores. Our scoring method differs in that it computes the *P *values of the match scores for the columns of the query motif aligned with a given target motif in all possible ways (without gaps). These 'offset' *P *values are computed using the cumulative density functions estimated from the target database, as described above. The minimum *P *value among these offset *P *values is used to compute the overall *P *value of the match between the query motif and the target motif, assuming independence of the offset *P *values. This is called the 'motif' *P *value. Finally, we apply a Bonferroni correction to the motif *P *values to derive an *E *value.

This algorithm is implemented in a software tool called Tomtom, which is publicly available as part of the MEME Suite of motif analysis tools [19-21]. Tomtom can compute *E *values based on any one of seven column comparison functions: PCC, ALLR, PCS, FIET, KLD, ED, or SW. In this work, we demonstrate the accuracy of Tomtom's statistical estimates. We also validate Tomtom'smotif retrieval accuracy via a simulation experiment. The results show that, in addition to providing formal semantics for motif similarity scores, Tomtom's *P *value estimation yields improved rankings relative to *ad hoc *normalization schemes.

## Results

### Algorithm

In this section, we describe the motif-motif comparison problem and outline our solution. Say we are given two motifs, *Q *and *T*. Our goal is to define a motif comparison function *S*(·,·), such that *S*(*Q*,*T*) is small if and only if *Q *and *T *resemble one another in some biologically relevant way. For now, let us sidestep the issue of defining 'biologically relevant' and assume that someone has given us a function *s*(·,·) that compares two motif columns. Thus, we can compare, for example, the *i*th column of *Q *and the *j*th column of *T *using *s*(*Q*_*i*_,*T*_*j*_). Our problem is to use the column comparison function *s*(·,·) to define the motif similarity function *S*(·,·).

This problem can be further subdivided into two subproblems. One subproblem is that we do not know *a priori *whether the motifs *Q *and *T *should be offset with respect to one another. Indeed, in the case of DNA motifs, we often do not even know whether the motifs lie on the same DNA strand. Therefore, our motif similarity function must take into account all possible offsets and relative orientations. A second subproblem is that even if we knew the correct offset and relative orientation, we need a method for combining the column comparison scores into a single score. Below, we describe solutions to each of these problems.

#### Computing offset *P *values

Initially, let us simplify our problem even further. Not only has someone told us the correct column-wise similarity function *s*(·,·), but they have also specified the correct relative offset and orientation of our motifs *Q *and *T*. For now, we assume that the motifs are of equal width *w*, that they lie in the same orientation, and that they have a relative offset of zero. Furthermore, we assume that columns of the motifs are independent and that our scores can be summed. Our problem is to compute a *P *value for this summation. Because the *P *value is relative to the given offset, we refer to this as the 'offset *P *value'. We adopt a dynamic programming method to calculate the null distribution of summed similarity scores with respect to the motif *Q*.

A similar method has been used to compute a *P *value for the match between a motif and a given sequence [16]. Briefly, that method can be described as follows. Say that we have a motif *Q *of width *w*, and we have a score function ŝ(*Q*_*i*_,*a*) that yields a positive integer score for the similarity of the *i*th column of *Q *and the letter *a *∈ *A*. These integral scores correspond to indices *x *of an array *A *defining the desired probability density function (PDF). *A *is filled recursively by noting that, if we know the PDF *A*^(*i*) ^for matches to the first *i *positions in *Q*, then we can calculate the PDF *A*^(*i*+1) ^as follows:

A(i+1)(x)=∑a∈AA(i)(x−S^
      
											[a,Qi+1])Pa     (1)
 MathType@MTEF@5@5@+=feaafiart1ev1aaatCvAUfeBSjuyZL2yd9gzLbvyNv2Caerbhv2BYDwAHbqedmvETj2BSbqee0evGueE0jxyaibaiKI8=vI8tuQ8FMI8Gi=hEeeu0xXdbba9frFj0=OqFfea0dXdd9vqai=hGuQ8kuc9pgc9s8qqaq=dirpe0xb9q8qiLsFr0=vr0=vr0dc8meaabaqaciGacaGaaeqabaqadeqadaaakeaacaWGbbWaaWbaaSqabeaacaGGOaGaamyAaiabgUcaRiaaigdacaGGPaaaaOGaaiikaiaadIhacaGGPaGaeyypa0ZaaabuaeaacaWGbbWaaWbaaSqabeaacaGGOaGaamyAaiaacMcaaaGccaGGOaGaamiEaiabgkHiTiqabofagaqcaiaabccacaGGBbGaamyyaiaacYcacaWGrbWaaSbaaSqaaiaadMgacqGHRaWkcaaIXaaabeaakiaac2facaGGPaGaamiuamaaBaaaleaacaWGHbaabeaaaeaacaWGHbGaeyicI4Saamyqaaqab0GaeyyeIuoakiaaxMaacaWLjaWaaeWaaeaacaaIXaaacaGLOaGaayzkaaaaaa@5528@

Where *P*_*a *_is the null probability of letter *a*. The recursion is initialized with *A*^(0)^(0) = 1 and A^(0)^(x) = 0 for *x *≥ 1. Iterating with *i *= 1 ... *w *yields the PDF for a random sequence matching the motif, which is used to calculate a cumulative probability distribution and thus *P *values. The challenge in generalizing the above algorithm to the motif-motif comparison problem arises because we do not have a fixed alphabet of amino acids or nucleotides for the summation in Eqn 1. Instead, we have an infinite 'alphabet' of motif columns. Our solution involves constructing an implicit alphabet of motif columns from the distribution of scores between all query motif columns versus all columns in a database of target motifs. This is an efficient solution because the matrix of query-versus-target motif column scores must be computed during the database search procedure.

In detail, the algorithm proceeds in five steps. First, for a given motif *Q *of width *w*_*Q *_and a given collection of target motifs *T*_1 _... *T*_*n *_whose total width is *w*_*T*_, we compute a *w*_*Q*_-by-*w*_*T *_matrix Γ such that Γ_*i*,*j *_= *s*(*Q*_*i*_,*T*_*j*_). This matrix constitutes the null distribution for our *P *value calculation. Second, we linearly rescale the values in Γ such that the minimum value is 1 and the maximum is *t*, where *t *is the (user-specified) number of letters in the motif column alphabet (in Tomtom, *t *= 100). We then round the values in Γ to integers. Third, for each column *i *in *Q *and for each possible scaled, integer score 1 ≤ *x *≤ *t*, we compute the frequency of *x *in the null distribution of the *i*th column of Γ:

fi,x=1wT∑j=1wTδ(Γi,j=x)     (2)
 MathType@MTEF@5@5@+=feaafiart1ev1aaatCvAUfeBSjuyZL2yd9gzLbvyNv2Caerbhv2BYDwAHbqedmvETj2BSbqee0evGueE0jxyaibaiKI8=vI8tuQ8FMI8Gi=hEeeu0xXdbba9frFj0=OqFfea0dXdd9vqai=hGuQ8kuc9pgc9s8qqaq=dirpe0xb9q8qiLsFr0=vr0=vr0dc8meaabaqaciGacaGaaeqabaqadeqadaaakeaacaWGMbWaaSbaaSqaaiaadMgacaGGSaGaamiEaaqabaGccqGH9aqpdaWcaaqaaiaaigdaaeaacaWG3bWaaSbaaSqaaiaadsfaaeqaaaaakmaaqahabaGaeqiTdqMaaiikaiabfo5ahnaaBaaaleaacaWGPbGaaiilaiaadQgaaeqaaOGaeyypa0JaamiEaiaacMcaaSqaaiaadQgacqGH9aqpcaaIXaaabaGaam4DamaaBaaameaacaWGubaabeaaa0GaeyyeIuoakiaaxMaacaWLjaWaaeWaaeaacaaIYaaacaGLOaGaayzkaaaaaa@4E78@

Where *δ*(·) is the Kronecker delta function. In the fourth step, we initialize a PDF *A*^(0)^, as described above, and then perform the recursion as follows:

A(i+1)(x)=∑y=1tA(i)(x−y)fi,y     (3)
 MathType@MTEF@5@5@+=feaafiart1ev1aaatCvAUfeBSjuyZL2yd9gzLbvyNv2Caerbhv2BYDwAHbqedmvETj2BSbqee0evGueE0jxyaibaiKI8=vI8tuQ8FMI8Gi=hEeeu0xXdbba9frFj0=OqFfea0dXdd9vqai=hGuQ8kuc9pgc9s8qqaq=dirpe0xb9q8qiLsFr0=vr0=vr0dc8meaabaqaciGacaGaaeqabaqadeqadaaakeaacaWGbbWaaWbaaSqabeaacaGGOaGaamyAaiabgUcaRiaaigdacaGGPaaaaOGaaiikaiaadIhacaGGPaGaeyypa0ZaaabCaeaacaWGbbWaaWbaaSqabeaacaGGOaGaamyAaiaacMcaaaGccaGGOaGaamiEaiabgkHiTiaadMhacaGGPaGaamOzamaaBaaaleaacaWGPbGaaiilaiaadMhaaeqaaaqaaiaadMhacqGH9aqpcaaIXaaabaGaamiDaaqdcqGHris5aOGaaCzcaiaaxMaadaqadaqaaiaaiodaaiaawIcacaGLPaaaaaa@5025@

**Figure 2:**
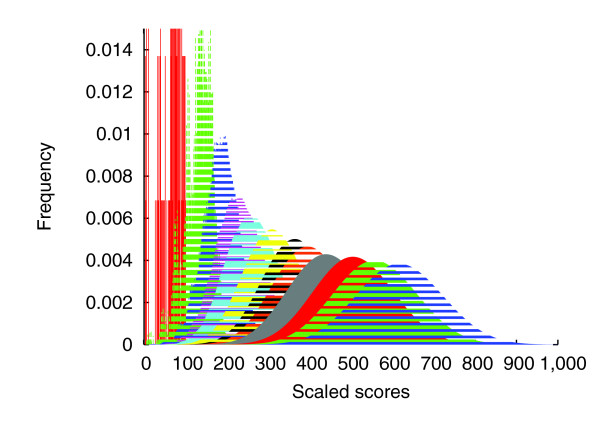
Score distribution histogram for a query motif of length 12. The figure contains 12 histograms overlaid on top of each other. Each histogram corresponds to the frequency distribution of scores, for an offset of zero relative to a query motif of width 12. The first (red) histogram is for the alignment involving only the first query column, the next (light green) histogram relates to the first two query columns, and so on.

The vectors *A*^(*i*) ^(1 ≤ *i *≤ *w*_*Q*_) contain the null PDFs of scaled, integerized scores for alignment to the first *i *columns of motif *Q*. In the fifth step, the PDF is converted to a cumulative density function, which can subsequently be used to compute offset *P *values. In a similar way, we derive PDFs for alignments starting and ending at arbitrary columns of the query. Figure 2 illustrates the output of the algorithm. The figure shows a set of PDFs for a particular query motif of length 12, computed relative to the TRANSFAC database. The figure contains 12 overlaid histograms, corresponding to different ways that the target might overlap with the query motif, assuming that the overlap begins at position 1. Any one of these histograms can be used to compute the *P *value of a score, depending on which columns of the query motif are aligned with the target motif. The corresponding *p *value is the area of the histogram to the right side of the computed score.

#### Computing motif *P *values

The above procedure yields a *P *value for a query and target motif with a particular offset and relative orientation. In order to compute a motif *P *value, Tomtom identifies the offset and relative orientation for which the offset *P *value is minimal. The probability of observing a minimum *P *value of P* among a collection of *N *independent *P *values is 1 - (1 - P*)^*N*^. This value is the motif *P *value.

#### Computing *E *values

Tomtom searches a target database of motifs using a given motif as the query. The resulting motif *P *values must therefore be corrected for multiple tests. Tomtom uses a form of Bonferroni correction that assumes that the targets are independent of one another. The correction consists of multiplying the motif *P *value by the number of targets in the database. The result is an *E *value - the expected number of times that the given query would be expected to match a target as well or better than the observed match in a randomized target database of the given size.

### Validation

We perform three separate experiments to assess the validity of Tomtom's statistical confidence estimates and measure Tomtom's ability to recognize related motifs.

#### Assessing *P *value accuracy

**Figure 3:**
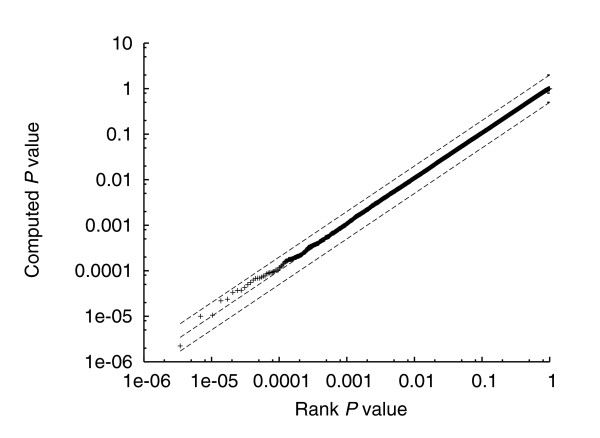
Accuracy of motif comparison *P *values. The figure plots the computed motif *P *value as a function of the empirical (rank-based) *P *value from searching shuffled query motifs against shuffled target motifs. The central line corresponds to *y *= *x*, and the two adjacent dotted lines correspond to *y *= 0.5*x *and *y *= 2*x*. The *P *values are computed using the euclidean distance.

To assess the accuracy of Tomtom's *P *value computation, we exploit the observation that null *P *values should be uniformly distributed between 0 and 1. We therefore generate a large quantity of *P *values using randomized data, and we measure the extent to which these *P *values are uniformly distributed. In order to generate null *P *values, we shuffle the columns of each motif in the TRANSFAC database (version 6.0) [5]. We then use Tomtom to search with a randomly selected query motif against the rest of the motifs in the target database. This iterative procedure, including shuffling, is repeated 1,000 times. The database contains 292 motifs, and so the entire procedure yields a total of 291,000 motif *P *values. We measure the uniformity of these *P *values using quantile-quantile plots, an example of which is shown in Figure 3. The figure plots the computed null *P *values against the theoretically correct, uniform distribution (so-called 'rank *P *values'). The dotted lines correspond to a twofold deviation from uniformity. This particular figure plots *P *values computed using the ED column similarity function; however, for all seven column similarity functions, the motif *P *values remain close to uniformity, rarely deviating by more than a factor of two (Additional data file 1). Note that in order for the motif *P *values to be accurate, the underlying offset *P *values must also be accurate. We verified (Additional data file 2) that the quantile-quantile plots for offset *P *values look similar to Figure 3.

#### Measuring retrieval accuracy

Next, we designed a simulation experiment to test Tomtom's ability to retrieve a related target motif from a database. The experiment is designed to simulate the following situation. Suppose that a researcher discovers a 'new' motif that is actually the same as one in a motif database. The new motif may contain some of the same sequences that were used to create the database motif plus some new sequences. Moreover, the exact boundaries of the novel motif may not exactly match the boundaries of the corresponding motif in the database. We simulate this situation, and then measure Tomtom's ability to identify the correct motif in the database.

In detail, the experiment proceeds as follows. We begin by selecting all 107 motifs in the JASPAR database (jaspar core). Then, we simulate a collection of 10 query and 10 target motifs for each of these JASPAR motifs by subsampling with replacement from the original sites of the JASPAR motif. The difficulty of the retrieval task can be modulated by reducing the number of sites sampled. In our first experiment, if the JASPAR motif has *S *associated sites, then the query and target motifs are simulated using *S*/2, *S*/4, *S*/8 or *S*/16 sites. In this step, we eliminate motifs that would yield fewer than two sampled sites, thus leaving 82 JASPAR motifs for the *S*/8 subsampling experiment. In the next step, we trim the edges of half of the motifs in each database. The number of columns to be deleted from a given motif is determined by selecting a random number uniformly from [-⌊0.8*w*⌋, ..., 0, 0, ..., ⌊0.8*w*⌋], where *w *is the motif width. The sign of the selected number determines which end of the motif is truncated. After this procedure, each motif in the JASPAR database has 10 corresponding motifs in the query and in the target database. Tomtom's task, given one of the query motifs, is to retrieve all 10 corresponding target motifs with smaller motif *P *values than any of the unrelated target motifs.

**Figure 4:**
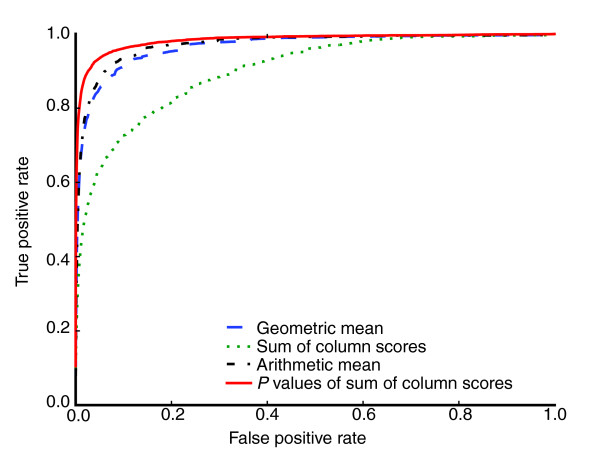
Measuring retrieval accuracy. Motif retrieval accuracy is estimated using simulated JASPAR motifs, as described in the text. The figure plots the percentage of correct query-target pairs (true positives) as a function of the percentage of incorrect pairs (false positives) as we traverse the list of query-target pairs sorted by Tomtom *P *value or any of the other three methods of combining column-wise scores. The solid and dashed lines correspond to width-normalized scores scores (*P *values, arithmetic mean, and geometric mean), and the green dotted line represents sum of column scores. This figure is for euclidean distance (ED) at a sampling rate of *S*/8.

We can use this simulation protocol to compare the various motif column comparison scores as well as to compare various methods of combining scores. Figure 4 compares four score combination methods: taking the sum of scores across all columns, the mean, the geometric mean, or the *P *value of the sum (as described under Algorithm, above). This figure is generated using ED as the scoring function and a sampling rate of *S*/8. The target database is ranked with respect to each query. Correct query-target pairs are labeled '+1' and incorrect pairs are labeled '-1'. All of the per-query lists are then sorted together into a single ranked list. From this ranked list, we compute a receiver operating characteristic (ROC) curve [23], which plots the percentage of positive pairs as a function of the percentage of negative pairs as we traverse the ranked list. The figure shows that the ranking produced by any normalization method is dramatically better than the ranking produced by the unnormalized sum. This is not surprising, because normalization aims to account for the difference in query motif lengths. The figure also suggests that, among the three normalization methods, *P *values yield a better ranking than the arithmetic or geometric mean. Similar results can be obtained using each of the other column comparison functions (Additional data file 5).

This result is encouraging; however, sorting the results from all of the queries into a single list is somewhat unrealistic. In practice, the user is only concerned with the quality of the ranking with respect to one motif at a time. We therefore compute ROC curves separately for each query motif. In order to quantify the extent to which the correct pairs appear near the top of the ranked list, we compute the area under each ROC curve (the ROC score). A perfect ranking would receive an ROC score of 1.0, whereas a random ranking would receive an ROC score of 0.5.

The resulting mean ROC scores are reported in Table 1. Surprisingly, regardless of the normalization method employed, the best performing column comparison function is the ED. Among the four ranking methods, the motif *P *value provides the best performance for five of the column comparison functions, and the sum of scores provides the best performance for the remaining two. Overall, the highest mean ROC score is achieved by motif *P *values using the ED. Notably, there is a significant improvement in the performance of 'Sum' as compared with results shown in Figure 4. The underlying data used to generate Table 1 and Figure 4 are the same; however, as noted above, in Table 1 a separate ROC score is computed for each motif and then a mean ROC score is computed, whereas in Figure 1 we rank all motifs together and compute a single ROC score. Because the motifs have varying lengths, the latter approach penalizes methods (such as 'Sum') that do not normalize for alignment length.

We conducted a statistical test to estimate the significance of the differences observed in Table 1. For each motif in the original database, we average the corresponding 10 ROC scores, yielding a list of 82 mean ROC scores for each column comparison method. We then compare the lists of mean ROC scores for two different methods using a signed rank test. The *P *values resulting from this analysis are summarized in Table 2. For the two competing normalization methods (arithmetic and geometric mean), Tomtom *P *values do a better job of combining column scores in every case. On the other hand, only one out of seven comparisons against the sum-of-scores method yields a significant difference. This lack of significance arises from the high variance in the mean ROCs produced by the summed column scores method. Overall, these results illustrate that Tomtom's *P *values reliably normalize for varying lengths of motif alignments, irrespective of the column comparison function used.

#### *E*-value based retrieval rate

In a third experiment, we tested the utility of the *E *values computed by Tomtom. This experiment was conducted to determine whether, using a reasonable significance threshold, Tomtom can successfully retrieve a JASPAR motif from the database. In this experiment, it is not sufficient for the correct target motif to have the best score; the score must also be statistically significant.

**Figure 5:**
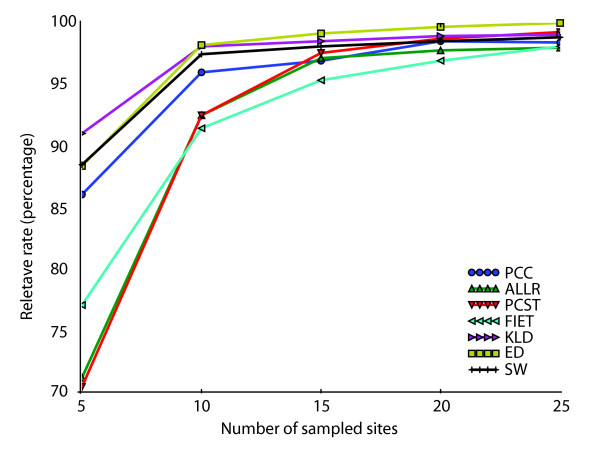
*E *value based retrieval rate. The figure plots the percentage of query motifs that successfully matched the correct JASPAR target as a function of the number of sites used to create the query motif. Here 'success' means that the top-ranked motif is the correct target and has an *E *value less than 0.01. ALLR, average log-likelihood ratio; ED, euclidean distance; FIET, Fisher-Irwin exact test; KLD, Kullback-Leibler divergence; PCC, Pearson correlation coefficient; PCST, Pearson *χ*^2 ^test; SW, Sandelin-Wasserman function.

As above, we simulate a collection 10 query motifs for each of the 100 JASPAR motifs that have at least 10 known sites. However, in this case, the target database is the original JASPAR database. Furthermore, unlike in the previous experiment, we sample a specific number of sites (5, 10, ..., 25) from each motif, rather than a fractional number of sites. This allows us to evaluate the effect of the number of sites on *E *value based retrieval rate. Tomtom is then used to compute *E *values for all 1,000 motifs in the query database. Finally, we compute the percentage of searches that are successful, where 'success' requires that the best *E *value match corresponds to the original JASPAR motif and that the corresponding *E *value is less than or equal to 0.01. Figure 5 plots the percentage of successful searches as a function of the number of sites sampled. With a query motif composed of five sites, the estimated probability of success is 92.7% using the best performing column comparison function. As expected, the retrieval rate increases with the increase in number of sites sampled; with 10 sampled sites the probability of success is 99.0%. The best performing motif column comparison functions are the ED and the KLD. Similar trends are observed using *E *value thresholds of 0.05 and 0.001 (Additional data file 6).

## Discussion

Tomtom is a motif comparison algorithm that ranks the target motifs in a given database according to the estimated statistical significance of the match between the query and the target. In this work we show that the motif *P *values computed by Tomtom are accurate, in the sense that they are uniformly distributed when computed on randomized data. We also show that the *P *value calculation produces rankings that are significantly better than the rankings produced by *ad hoc *normalization schemes. It is important to emphasize, however, that even if the rankings produced by Tomtom were no better than *ad hoc *rankings produced, *P *value normalization would still be the preferred method because of the inherent advantages of having a measure of the statistical significance of query-target matches. Finally, we show that Tomtom correctly assigns *E *values less than 0.01 to a large percentage of positive matches. This result indicates that it is highly probable that Tomtom successfully retrieves a related motif with a significant *E *value. All of these properties make Tomtom a valuable tool for identifying truly related motifs.

During the course of our experiments, we compared seven different motif column comparison functions. Surprisingly, the simple ED between motif columns performs best. Consequently, Tomtom's default behavior is to compare columns using ED. However, for some types of motifs (for instance, protein motifs) other comparison functions may be more appropriate. Consequently, Tomtom provides an option to use any of the seven column comparison functions.

In terms of practical applicability, Tomtom is especially relevant in conjunction with MEME, an *ab initio *motif discovery tool. Novel motifs identified using MEME can be reliably searched against known motifs using Tomtom. Both Tomtom and MEME are currently available as part of the MEME Suite of motif analysis tools [19,20], and a Tomtom website is under development.

## Materials and methods

### Motif column comparison functions

At Tomtom's core is a function that defines the similarity between one column of the query and one column of the target motif. Tomtom implements seven such functions, described below. The 'raw' score for an ungapped alignment of columns from a query motif and a target motif is computed by summing the column comparison scores computed using any of the following functions. Tomtom converts the raw scores into *P *values and *E *values, as described above.

In the following discussion, *X *refers to a column of the query motif, and is a multinomial probability vector. The quantity *X*_*a *_refers to the probability of letter *a *∈ *A *in *X*. For some of these functions, these probabilities are multiplied by a motif-dependent constant to give the 'counts' of different letters in each column of the motif. We use *N*_*Xa *_to refer to the count of letter *a *in column *X*. Similar definitions apply for *Y*, a column from the target motif. The quantity |*A*| refers to the length of the motif alphabet (four for DNA, 20 for proteins).

### Pearson correlation coefficient

The PCC was first introduced for computing motif-motif similarity by Pietrokovski [7]. For two columns *X *and *Y*, PCC is computed using the following formula:

PCC(X,Y)=∑a∈A(Xa−X¯)(Yb−Y¯)∑a∈A(Xa−X¯)2∑a∈A(Ya−Y¯)2),X¯=1|A|∑a∈AXaY¯=1|A|∑a∈AYa
 MathType@MTEF@5@5@+=feaafiart1ev1aaatCvAUfeBSjuyZL2yd9gzLbvyNv2Caerbhv2BYDwAHbqedmvETj2BSbqee0evGueE0jxyaibaiKI8=vI8tuQ8FMI8Gi=hEeeu0xXdbba9frFj0=OqFfea0dXdd9vqai=hGuQ8kuc9pgc9s8qqaq=dirpe0xb9q8qiLsFr0=vr0=vr0dc8meaabaqaciGacaGaaeqabaqadeqadaaakqaabeqaaiaabcfacaqGdbGaae4qaiaacIcacaWGybGaaiilaiaadMfacaGGPaGaeyypa0ZaaSaaaeaadaaeqbqaamaabmaabaGaamiwamaaBaaaleaacaWGHbaabeaakiabgkHiTiqadIfagaqeaaGaayjkaiaawMcaamaabmaabaGaamywamaaBaaaleaacaWGIbaabeaakiabgkHiTiqadMfagaqeaaGaayjkaiaawMcaaaWcbaGaamyyaiabgIGioprtHrhAL1wy0L2yHvtyaeHbnfgDOvwBHrxAJfwnaGabaiab=bq8bbqab0GaeyyeIuoaaOqaamaakeaabaWaaabuaeaadaqadaqaaiaadIfadaWgaaWcbaGaamyyaaqabaGccqGHsislceWGybGbaebaaiaawIcacaGLPaaadaahaaWcbeqaaiaaikdaaaaabaGaamyyaiabgIGiolab=bq8bbqab0GaeyyeIuoakmaaqafabaWaaeWaaeaacaWGzbWaaSbaaSqaaiaadggaaeqaaOGaeyOeI0IabmywayaaraaacaGLOaGaayzkaaWaaWbaaSqabeaacaaIYaaaaaqaaiaadggacqGHiiIZcqWFaeFqaeqaniabggHiLdaaleqaaaaakiaacMcacaGGSaaabaGabmiwayaaraGaeyypa0ZaaSaaaeaacaaIXaaabaGaaiiFaiab=bq8bjaacYhaaaWaaabuaeaacaWGybWaaSbaaSqaaiaadggaaeqaaaqaaiaadggacqGHiiIZcqWFaeFqaeqaniabggHiLdaakeaaceWGzbGbaebacqGH9aqpdaWcaaqaaiaaigdaaeaacaGG8bGae8haXhKaaiiFaaaadaaeqbqaaiaadMfadaWgaaWcbaGaamyyaaqabaaabaGaamyyaiabgIGiolab=bq8bbqab0GaeyyeIuoaaaaa@8D35@

The latter two expressions reduce to 1|A|
 MathType@MTEF@5@5@+=feaafiart1ev1aaatCvAUfeBSjuyZL2yd9gzLbvyNv2Caerbhv2BYDwAHbqedmvETj2BSbqee0evGueE0jxyaibaiKI8=vI8tuQ8FMI8Gi=hEeeu0xXdbba9frFj0=OqFfea0dXdd9vqai=hGuQ8kuc9pgc9s8qqaq=dirpe0xb9q8qiLsFr0=vr0=vr0dc8meaabaqaciGacaGaaeqabaqadeqadaaakeaadaWcaaqaaiaaigdaaeaacaGG8bGaamyqaiaacYhaaaaaaa@36BE@, because

∑a∈AXa=∑a∈AYa=1
 MathType@MTEF@5@5@+=feaafiart1ev1aaatCvAUfeBSjuyZL2yd9gzLbvyNv2Caerbhv2BYDwAHbqedmvETj2BSbqee0evGueE0jxyaibaiKI8=vI8tuQ8FMI8Gi=hEeeu0xXdbba9frFj0=OqFfea0dXdd9vqai=hGuQ8kuc9pgc9s8qqaq=dirpe0xb9q8qiLsFr0=vr0=vr0dc8meaabaqaciGacaGaaeqabaqadeqadaaakeaadaaeqbqaaiaadIfadaWgaaWcbaGaamyyaaqabaaabaGaamyyaiabgIGioprtHrhAL1wy0L2yHvtyaeHbnfgDOvwBHrxAJfwnaGabaiab=bq8bbqab0GaeyyeIuoakiabg2da9maaqafabaGaamywamaaBaaaleaacaWGHbaabeaaaeaacaWGHbGaeyicI4Sae8haXheabeqdcqGHris5aOGaeyypa0JaaGymaaaa@5016@

for multinomial probability vectors *X *and *Y*.

### Average log-likelihood ratio

The ALLR formula described by Wang and Stormo [9] to quantify similarity between columns *X *and *Y *for position specific weight matrix motifs is as follows:

ALLR(X,Y)=∑a∈ANXalog(Yapa)+∑a∈ANYalog(Xapa)∑a∈A(NXa+NYa),
 MathType@MTEF@5@5@+=feaafiart1ev1aaatCvAUfeBSjuyZL2yd9gzLbvyNv2Caerbhv2BYDwAHbqedmvETj2BSbqee0evGueE0jxyaibaiKI8=vI8tuQ8FMI8Gi=hEeeu0xXdbba9frFj0=OqFfea0dXdd9vqai=hGuQ8kuc9pgc9s8qqaq=dirpe0xb9q8qiLsFr0=vr0=vr0dc8meaabaqaciGacaGaaeqabaqadeqadaaakeaacaqGbbGaaeitaiaabYeacaqGsbGaaiikaiaadIfacaGGSaGaamywaiaacMcacqGH9aqpdaWcaaqaamaaqafabaGaamOtamaaBaaaleaacaWGybGaamyyaaqabaGccaqGSbGaae4BaiaabEgadaqadaqaamaalaaabaGaamywamaaBaaaleaacaWGHbaabeaaaOqaaiaadchadaWgaaWcbaGaamyyaaqabaaaaaGccaGLOaGaayzkaaaaleaacaWGHbGaeyicI48enfgDOvwBHrxAJfwnHbqeg0uy0HwzTfgDPnwy1aaceaGae8haXheabeqdcqGHris5aOGaey4kaSYaaabuaeaacaWGobWaaSbaaSqaaiaadMfacaWGHbaabeaakiaabYgacaqGVbGaae4zamaabmaabaWaaSaaaeaacaWGybWaaSbaaSqaaiaadggaaeqaaaGcbaGaamiCamaaBaaaleaacaWGHbaabeaaaaaakiaawIcacaGLPaaaaSqaaiaadggacqGHiiIZcqWFaeFqaeqaniabggHiLdaakeaadaaeqbqaaiaacIcacaWGobWaaSbaaSqaaiaadIfacaWGHbaabeaakiabgUcaRiaad6eadaWgaaWcbaGaamywaiaadggaaeqaaOGaaiykaaWcbaGaamyyaiabgIGiolab=bq8bbqab0GaeyyeIuoaaaGccaGGSaaaaa@77C1@

where *P*_*a *_is the background (prior) frequency of letter *a*.

### Pearson *χ*^2 ^test

The Pearson *χ*^2 ^test was introduced by Schones and coworkers [10] for comparing motifs. The *χ*^2 ^*P *value is computed for the null hypothesis that the aligned columns are independent and identically distributed observations from the same multinomial distribution. In order to compute the value of *χ*^2^, a contingency table with margins is constructed (Table 3). Using the contingency table, the value of *χ*^2 ^is computed using the following equation:

χ2(X,Y)=∑j=X,Y∑a∈A(Njae−Njao)2Njae
 MathType@MTEF@5@5@+=feaafiart1ev1aaatCvAUfeBSjuyZL2yd9gzLbvyNv2Caerbhv2BYDwAHbqedmvETj2BSbqee0evGueE0jxyaibaiKI8=vI8tuQ8FMI8Gi=hEeeu0xXdbba9frFj0=OqFfea0dXdd9vqai=hGuQ8kuc9pgc9s8qqaq=dirpe0xb9q8qiLsFr0=vr0=vr0dc8meaabaqaciGacaGaaeqabaqadeqadaaakeaacqaHhpWydaahaaWcbeqaaiaaikdaaaGccaGGOaGaamiwaiaacYcacaWGzbGaaiykaiabg2da9maaqafabaWaaabuaeaadaWcaaqaaiaacIcacaWGobWaa0baaSqaaiaadQgacaWGHbaabaGaamyzaaaakiabgkHiTiaad6eadaqhaaWcbaGaamOAaiaadggaaeaacaWGVbaaaOGaaiykamaaCaaaleqabaGaaGOmaaaaaOqaaiaad6eadaqhaaWcbaGaamOAaiaadggaaeaacaWGLbaaaaaaaeaacaWGHbGaeyicI4Saamyqaaqab0GaeyyeIuoaaSqaaiaadQgacqGH9aqpcaWGybGaaiilaiaadMfaaeqaniabggHiLdaaaa@5510@

Where *N*^*o*^_*ja *_= *N*_*ja *_is the 'observed' count of letter *a *in column *j*, and *N*^*e*^_*ja *_= *N*_*j*_*N*_*a*_/*N *is the 'expected' count of letter *a *at column *j *(Table 3 for definitions).

The *P *value is calculated from this *χ*^2 ^score using |*A*| - 1 degrees of freedom. Because our null hypothesis is that these two columns are derived from the same multinomial distribution, a higher *P *value implies similarity. This *P *value is treated as an additive score.

### Fisher-Irwin exact test

The FIET [10] is an analytical computation of the Pearson *χ*^2 ^*P *value. In particular, this calculation is important when marginal frequencies are small, which is often the case in position frequency matrices. The marginal *P *value of the contingency table for DNA motifs (Table 3) follows the multiple hypergeometric distribution [24]:

P=NXNXA,NXC,NXG,NXTNYNYA,NYC,NYG,NYTN NA,NC,NG,NT
 MathType@MTEF@5@5@+=feaafiart1ev1aaatCvAUfeBSjuyZL2yd9gzLbvyNv2Caerbhv2BYDwAHbqedmvETj2BSbqee0evGueE0jxyaibaiKI8=vI8tuQ8FMI8Gi=hEeeu0xXdbba9frFj0=OqFfea0dXdd9vqai=hGuQ8kuc9pgc9s8qqaq=dirpe0xb9q8qiLsFr0=vr0=vr0dc8meaabaqaciGacaGaaeqabaqadeqadaaakeaacaWGqbGaeyypa0ZaaSaaaeaacaWGobWaaSbaaSqaaiaadIfaaeqaaOGaamOtamaaBaaaleaacaWGybGaamyqaaqabaGccaGGSaGaamOtamaaBaaaleaacaWGybGaam4qaaqabaGccaGGSaGaamOtamaaBaaaleaacaWGybGaam4raaqabaGccaGGSaGaamOtamaaBaaaleaacaWGybGaamivaaqabaGccaWGobWaaSbaaSqaaiaadMfaaeqaaOGaamOtamaaBaaaleaacaWGzbGaamyqaaqabaGccaGGSaGaamOtamaaBaaaleaacaWGzbGaam4qaaqabaGccaGGSaGaamOtamaaBaaaleaacaWGzbGaam4raaqabaGccaGGSaGaamOtamaaBaaaleaacaWGzbGaamivaaqabaaakeaacaWGobGaaeiiaiaad6eadaWgaaWcbaGaamyqaaqabaGccaGGSaGaamOtamaaBaaaleaacaWGdbaabeaakiaacYcacaWGobWaaSbaaSqaaiaadEeaaeqaaOGaaiilaiaad6eadaWgaaWcbaGaamivaaqabaaaaaaa@5D72@

The formula for protein motifs is similar. The two-sided *P *value for the table is the sum of probabilities of all tables that are at least as extreme. This *P *value is computed using the algorithm described by Mehta and Patel [25]. As with the *χ*^2 ^test, this *P *value is used as an additive score.

### Kullback-Leibler divergence

The KLD has been used by several research groups to quantify similarity between motifs [11-13]. The symmetric form of KLD for two columns X and Y is given by the following equation:

KLD(X,Y)=12(∑a∈AXalog(XaYa)+∑a∈AYalog(YaXa)).
 MathType@MTEF@5@5@+=feaafiart1ev1aaatCvAUfeBSjuyZL2yd9gzLbvyNv2Caerbhv2BYDwAHbqedmvETj2BSbqee0evGueE0jxyaibaiKI8=vI8tuQ8FMI8Gi=hEeeu0xXdbba9frFj0=OqFfea0dXdd9vqai=hGuQ8kuc9pgc9s8qqaq=dirpe0xb9q8qiLsFr0=vr0=vr0dc8meaabaqaciGacaGaaeqabaqadeqadaaakeaacaqGlbGaaeitaiaabseacaGGOaGaamiwaiaacYcacaWGzbGaaiykaiabg2da9maalaaabaGaaGymaaqaaiaaikdaaaWaaeWaaeaadaaeqbqaaiaadIfadaWgaaWcbaGaamyyaaqabaacbaGccaWFSbGaa83Baiaa=DgadaqadaqaamaalaaabaGaamiwamaaBaaaleaacaWGHbaabeaaaOqaaiaadMfadaWgaaWcbaGaamyyaaqabaaaaaGccaGLOaGaayzkaaaaleaacaWGHbGaeyicI48enfgDOvwBHrxAJfwnHbqeg0uy0HwzTfgDPnwy1aaceaGae4haXheabeqdcqGHris5aOGaey4kaSYaaabuaeaacaWGzbWaaSbaaSqaaiaadggaaeqaaOGaa8hBaiaa=9gacaWFNbWaaeWaaeaadaWcaaqaaiaadMfadaWgaaWcbaGaamyyaaqabaaakeaacaWGybWaaSbaaSqaaiaadggaaeqaaaaaaOGaayjkaiaawMcaaaWcbaGaamyyaiabgIGiolab+bq8bbqab0GaeyyeIuoaaOGaayjkaiaawMcaaiaac6caaaa@69F4@

### Euclidean distance

Choi and coworkers [14] introduced the ED as a means to compare protein motifs. The ED for two DNA profile columns X and Y is computed using the following formula:

ED(X,Y)=−∑a∈A(Xa−Ya)2.
 MathType@MTEF@5@5@+=feaafiart1ev1aaatCvAUfeBSjuyZL2yd9gzLbvyNv2Caerbhv2BYDwAHbqedmvETj2BSbqee0evGueE0jxyaibaiKI8=vI8tuQ8FMI8Gi=hEeeu0xXdbba9frFj0=OqFfea0dXdd9vqai=hGuQ8kuc9pgc9s8qqaq=dirpe0xb9q8qiLsFr0=vr0=vr0dc8meaabaqaciGacaGaaeqabaqadeqadaaakeaacaqGfbGaaeiraiaacIcacaWGybGaaiilaiaadMfacaGGPaGaeyypa0JaeyOeI0YaaOqaaeaadaaeqbqaamaabmaabaGaamiwamaaBaaaleaacaWGHbaabeaakiabgkHiTiaadMfadaWgaaWcbaGaamyyaaqabaaakiaawIcacaGLPaaadaahaaWcbeqaaiaaikdaaaaabaGaamyyaiabgIGioprtHrhAL1wy0L2yHvtyaeHbnfgDOvwBHrxAJfwnaGabaiab=bq8bbqab0GaeyyeIuoaaSqabaGccaGGUaaaaa@5293@

### Sandelin-Wasserman similarity function

Sandelin and Wasserman [15] introduced their own motif column comparison function for the construction of familial binding profiles. The SW score for two columns X and Y is computed using the following formula:

SW(X,Y)=2−∑a∈A(Xa−Ya)2.
 MathType@MTEF@5@5@+=feaafiart1ev1aaatCvAUfeBSjuyZL2yd9gzLbvyNv2Caerbhv2BYDwAHbqedmvETj2BSbqee0evGueE0jxyaibaiKI8=vI8tuQ8FMI8Gi=hEeeu0xXdbba9frFj0=OqFfea0dXdd9vqai=hGuQ8kuc9pgc9s8qqaq=dirpe0xb9q8qiLsFr0=vr0=vr0dc8meaabaqaciGacaGaaeqabaqadeqadaaakeaacaqGtbGaae4vaiaacIcacaWGybGaaiilaiaadMfacaGGPaGaeyypa0JaaGOmaiabgkHiTmaaqafabaWaaeWaaeaacaWGybWaaSbaaSqaaiaadggaaeqaaOGaeyOeI0IaamywamaaBaaaleaacaWGHbaabeaaaOGaayjkaiaawMcaamaaCaaaleqabaGaaGOmaaaaaeaacaWGHbGaeyicI48enfgDOvwBHrxAJfwnHbqeg0uy0HwzTfgDPnwy1aaceaGae8haXheabeqdcqGHris5aOGaaiOlaaaa@5354@

## Authors' contributions

JS, TB, and WSN initially conceptualized the project. TB and WSN conceptualized the dynamic programming framework of Tomtom. TB suggested the method for calculating *P *values by using observed columns in the motif database. WSN and SG were responsible for the detailed design of the program. SG implemented the program as a part of the MEME Suite, relying on code previously written by TB and WSN. TB suggested the simulation experiment using sampling of actual motif sites, and the *E *value retrieval rate for evaluating search methods. WSN, TB and SG designed the various simulation experiments that were performed by SG. SG and WSN drafted the manuscript, with revisions from TB. All authors read and approved the final manuscript.

## Additional data files

The following additional data are available with the online version of this paper. Additional data file 1 is a figure showing the accuracy of motif comparison *P *values. Additional data file 2 is a figure showing the accuracy of offset *P *values. Additional data file 3 is a table summarizing the mean ROCs for various motif column comparison functions and score combination methods for various sampling rates. Additional data file 4 is a table comparing motif *P *values with other methods of combining column scores for various sampling rates. Additional data file 5 is a figure showing the motif retrieval accuracy for various column similarity functions at a sampling rate of *S*/8. Additional data file 6 is a figure whosing the *E *value based retrieval rate for two additional significance levels (*E *value less than 0.05 or 0.001).

## Supplementary Material

Additional data file 1 Accuracy of motif comparison *P *values: QQ plots for various column comparison functions Click here for file

Additional data file 2 Accuracy of offset *P *values: QQ plots for various column comparison functions Click here for file

Additional data file 3 Mean ROC scores for various motif column comparison functions and score combination methods for various sampling rates Click here for file

Additional data file 4 Comparison of motif *P *values versus other methods of combining column scores for various sampling rates Click here for file

Additional data file 5 Motif retrieval accuracy for various column similarity functions at a sampling rate of *S*/8 Click here for file

Additional data file 6 *E *value based retrieval rate for two additional significance levels (*E*-value less than 0.05 or 0.001) Click here for file

## Figures and Tables

**Table 1:** Mean ROC scores for various motif column comparison functions and score combination methods

Ranking method	ALLR	PCC	PCST	FIET	KLD	ED	SW
Sum	0.9823^a^	0.9845^a^	0.9786	0.9834	0.9793	0.9886	0.9809
AM	0.9595	0.9685	0.9619	0.9662	0.9736	0.9779	0.9735
GM	0.9643	0.9670	0.9630	0.9717	0.9724	0.9776	0.9720
*p *value	0.9786	0.9835	0.9797^a^	0.9842^a^	0.9864^a^	0.9889^a^	0.9861^a^

The table reports the performance of the seven different column comparison functions using four different methods for combining scores: summing the raw scores, computing the arithmetic mean (AM), or computing the geometric mean (GM). Each entry is the mean receiver operating characteristic (ROC) score across all queries in the simulation. The table reports results for the *S*/8 sampling rate. ^a^Highest ROC in the column. ALLR, average log-likelihood ratio; ED, euclidean distance; FIET, Fisher-Irwin exact test; KLD, Kullback-Leibler divergence; PCC, Pearson correlation coefficient; PCST, Pearson *χ*^2 ^test; SW, Sandelin-Wasserman function.

**Table 2:** Comparison of motif *P *values with other methods of combining column scores

	ALLR	PCC	PCST	FIET	KLD	ED	SW
*P *value versus sum	-	-	-	-	1.87 × e-03	-	-
*P *value versus AM	3.55 × e^-13^	2.43 × e^-13^	2.35 × e^-13^	6.93 × e^-14^	2.05 × e^-13^	1.38 × e^-12^	2.19 × e^-13^
*P *value versus GM	7.00 × e^-11^	2.52 × e^-13^	2.04 × e^-12^	2.06 × e^-10^	1.09 × e^-13^	1.58 × e^-12^	1.98 × e^-13^

The table compares the performance of Tomtom's *P *values with three other methods for combining column motif comparison scores: summing the raw scores (sum), computing the arithmetic mean (AM), and computing the geometric mean (GM). The comparison is performed for seven different column comparison functions. Each entry in the table is a signed rank *P *value for the comparison of two ranking methods. '-' Indicates that the difference between the two methods is not significant at *P *= 0.01. All entries correspond to significantly better performance of motif *P *values than the competing method (Table 1). The table reports results for the *S*/8 sampling rate. ALLR, average log-likelihood ratio; ED, euclidean distance; FIET, Fisher-Irwin exact test; KLD, Kullback-Leibler divergence; PCC, Pearson correlation coefficient; PCST, Pearson *χ*^2 ^test; SW, Sandelin-Wasserman function.

**Table 3:** Contingency table (with margins) for DNA motifs

	A	C	G	T	-
*X*	*N*_*XA*_	*N*_*XC*_	*N*_*XG*_	*N*_*XT*_	*N*_*X*_
*Y*	*N*_*YA*_	*N*_*YC*_	*N*_*YG*_	*N*_*YT*_	*N*_*Y*_

	*N*_*A*_	*N*_*C*_	*N*_*G*_	*N*_*T*_	*N*

This table is used for computing columnwise scores for Pearson *χ*^2 ^and for Fisher-Irwin exact test. A similar table with 20 columns is used for protein motifs.
