# Supplementary material for: Quantifying similarity between motifs
Source: Genome Biol. 2007 Feb 26;8(2):R24. doi: 10.1186/gb-2007-8-2-r24 (PMC1852410; doi:10.1186/gb-2007-8-2-r24)
Supplement: Additional data file 2 — Accuracy of offset P values: QQ plots for various column comparison functions [file gb-2007-8-2-r24-S2.pdf]

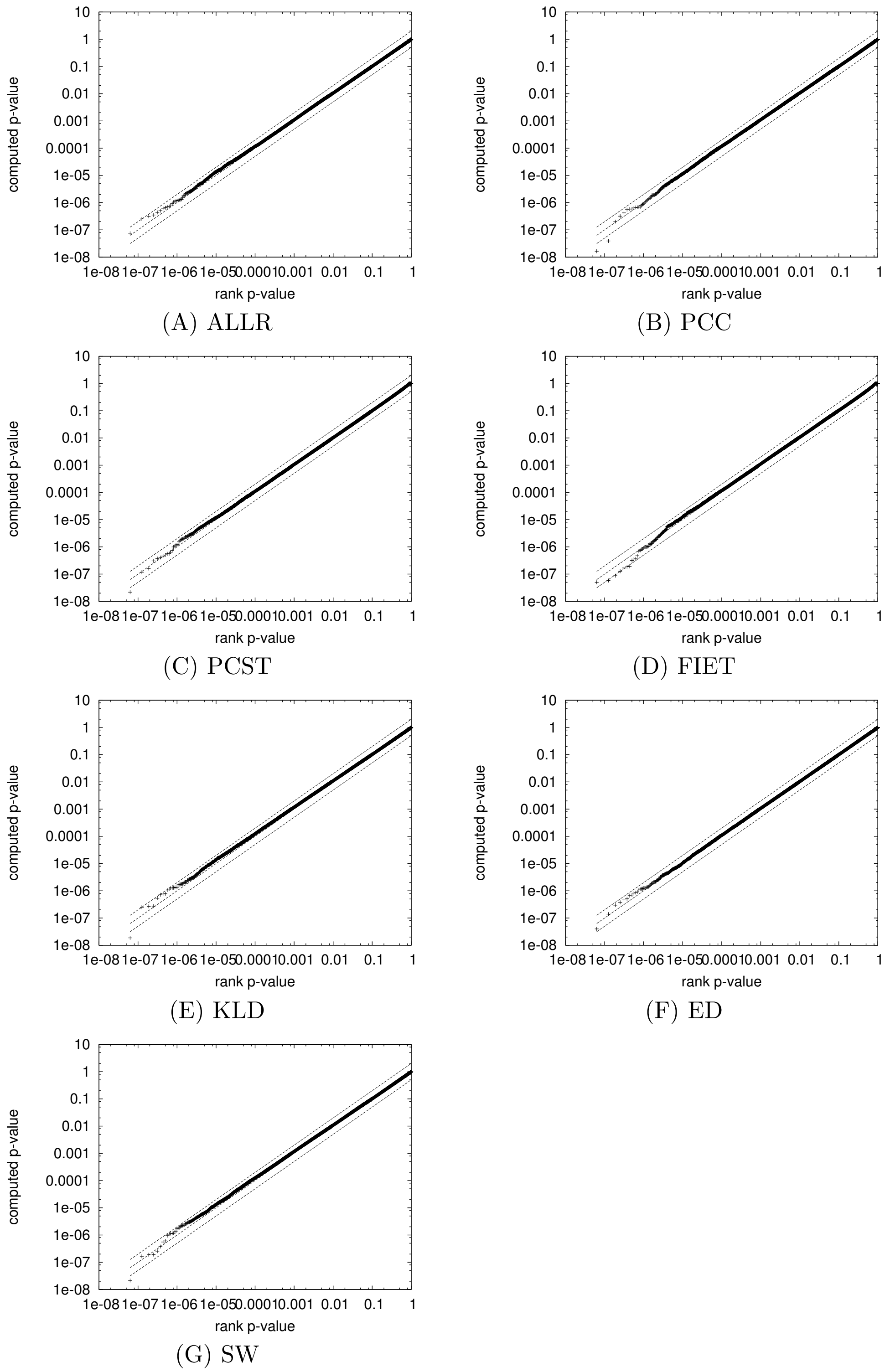

Figure 2: **Accuracy of offset p-values.** Each figure plots the computed offset p-value as a function of the empirical (rank-based) p-value from searching shuffled query motifs against shuffled target motifs. The central line corresponds $y = x$, while the two adjacent dotted lines correspond to $y = 0.5x$ and $y = 2x$. Each panel contains p-values computed using a different column similarity function.
