# Supplementary material for: Quantifying similarity between motifs
Source: Genome Biol. 2007 Feb 26;8(2):R24. doi: 10.1186/gb-2007-8-2-r24 (PMC1852410; doi:10.1186/gb-2007-8-2-r24)
Supplement: Additional data file 3 — Mean ROC scores for various motif column comparison functions and score combination methods for various sampling rates [file gb-2007-8-2-r24-S3.pdf]

A) $S/2$ sampling rate

Ranking method	ALLR	PCC	PCST	FIET	KLD	ED	SW
Sum	0.9909	0.9928	0.9943	0.9928	0.9855	0.9945	0.9888
AM	0.9837	0.9892	0.9904	0.9872	0.9926	0.9923	0.9913
GM	0.9856	0.9890	0.9926	0.9930	0.9924	0.9921	0.9911
p-value	**0.9934**	**0.9951**	**0.9958**	**0.9940**	**0.9965**	**0.9967**	**0.9960**

B) $S/4$ sampling rate

Ranking method	ALLR	PCC	PCST	FIET	KLD	ED	SW
Sum	**0.9875**	**0.9895**	0.9883	0.9892	0.9818	0.9922	0.9849
AM	0.9660	0.9774	0.9789	0.9786	0.9840	0.9859	0.9820
GM	0.9733	0.9768	0.9811	0.9848	0.9833	0.9849	0.9810
p-value	0.9823	0.9890	**0.9895**	**0.9894**	**0.9927**	**0.9935**	**0.9916**

C) $S/8$ sampling rate

Ranking method	ALLR	PCC	PCST	FIET	KLD	ED	SW
Sum	**0.9823**	**0.9845**	0.9786	0.9834	0.9793	0.9886	0.9809
AM	0.9595	0.9685	0.9619	0.9662	0.9736	0.9779	0.9735
GM	0.9643	0.9670	0.9630	0.9717	0.9724	0.9776	0.9720
p-value	0.9786	0.9835	**0.9797**	**0.9842**	**0.9864**	**0.9889**	**0.9861**

D) $S/16$ sampling rate

Ranking method	ALLR	PCC	PCST	FIET	KLD	ED	SW
Sum	0.9832	0.9842	0.9717	0.9794	0.9799	0.9864	0.9807
AM	0.9629	0.9684	0.9561	0.9627	0.9703	0.9724	0.9714
GM	0.9646	0.9658	0.9566	0.9650	0.9689	0.9745	0.9689
p-value	**0.9835**	**0.9856**	**0.9778**	**0.9863**	**0.9865**	**0.9877**	**0.9871**

Table 1: **Mean ROCs for various motif column comparison functions and score combination methods.** The table reports the performance of the seven different column comparison functions using four different methods for combining scores: summing the raw scores, computing the arithmetic mean (AM), or computing the geometric mean (GM). Each entry is the mean ROC score across all queries in the simulation. Bold font indicates the highest ROC in each column. The four panels in the table report results for four different sampling rates.
