# Supplementary material for: Quantifying similarity between motifs
Source: Genome Biol. 2007 Feb 26;8(2):R24. doi: 10.1186/gb-2007-8-2-r24 (PMC1852410; doi:10.1186/gb-2007-8-2-r24)
Supplement: Additional data file 4 — Comparison of motif P values versus other methods of combining column scores for various sampling rates [file gb-2007-8-2-r24-S4.pdf]

A) $S/2$ sampling rate

Ranking method	ALLR	PCC	PCST	FIET	KLD	ED	SW
p-value vs. sum	6.43e-04	2.63e-05	—	—	2.22e-16	3.09e-05	3.24e-14
p-value vs. AM	6.66e-16	2.22e-16	0.00e+00	0.00e+00	3.55e-15	2.22e-15	8.88e-16
p-value vs. GM	8.93e-14	6.66e-16	6.65e-08	—	4.44e-15	1.47e-14	1.11e-15

B) $S/4$ sampling rate

Ranking method	ALLR	PCC	PCST	FIET	KLD	ED	SW
p-value vs. sum	—	—	—	—	4.09e-10	—	1.03e-06
p-value vs. AM	3.55e-15	6.66e-16	0.00e+00	0.00e+00	6.66e-16	2.00e-15	4.44e-16
p-value vs. GM	6.00e-12	1.78e-15	4.06e-13	3.19e-07	4.44e-16	2.22e-15	2.22e-16

C) $S/8$ sampling rate

Ranking method	ALLR	PCC	PCST	FIET	KLD	ED	SW
p-value vs. sum	—	—	—	—	1.87e-03	—	—
p-value vs. AM	3.55e-13	2.43e-13	2.35e-13	6.93e-14	2.05e-13	1.38e-12	2.19e-13
p-value vs. GM	7.00e-11	2.52e-13	2.04e-12	2.06e-10	1.09e-13	1.58e-12	1.98e-13

D) $S/16$ sampling rate

Ranking method	ALLR	PCC	PCST	FIET	KLD	ED	SW
p-value vs. sum	—	—	—	—	—	—	—
p-value vs. AM	5.93e-07	8.09e-07	1.52e-07	1.52e-07	4.68e-07	4.00e-07	9.08e-07
p-value vs. GM	7.20e-07	5.07e-07	1.65e-07	4.00e-07	3.15e-07	2.01e-06	4.00e-07

Table 2: **Comparison of motif p-values versus other methods of combining column scores.** The table compares the performance of Tomtom's p-values with three other methods for combining column motif comparison scores: summing the raw scores, computing the arithmetic mean (AM), or computing the geometric mean (GM). The comparison is performed for seven different column comparison functions. Each entry in the table is a signed rank p-value for the comparison of two ranking methods. A '—' indicates that the difference between the two methods is not significant at $p = 0.01$. All entries correspond to significantly better performance of motif p-values than the competing method. The four panels report results for the different sampling rates.
