# Supplementary material for: Quantifying similarity between motifs
Source: Genome Biol. 2007 Feb 26;8(2):R24. doi: 10.1186/gb-2007-8-2-r24 (PMC1852410; doi:10.1186/gb-2007-8-2-r24)
Supplement: Additional data file 5 — Motif retrieval accuracy for various column similarity functions at a sampling rate of S/8 [file gb-2007-8-2-r24-S5.pdf]

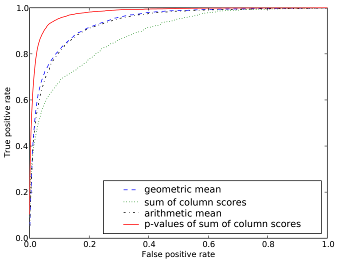


(A) ALLR

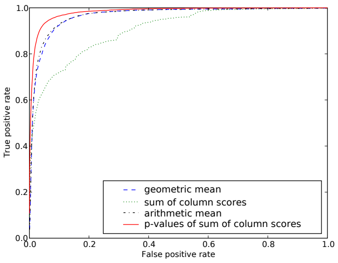


(B) PCC

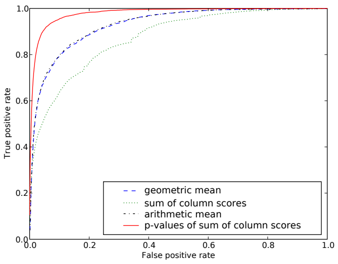


(C) PCST

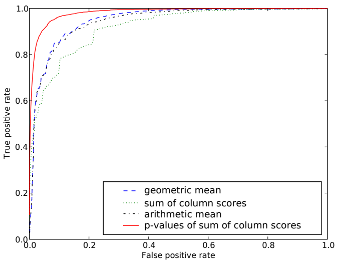


(D) FIET

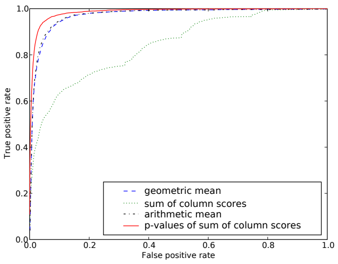


(D) KLD

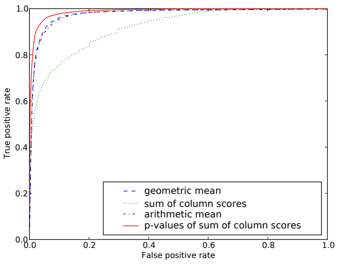


(E) ED

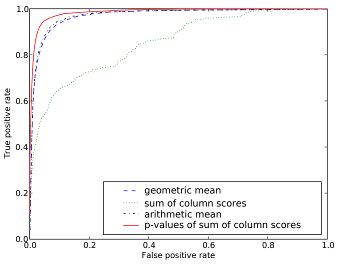


(F) SW

Figure 3: **Measuring retrieval accuracy.** Motif retrieval accuracy is estimated using simulated JASPAR motifs, as described in the text. The figure plots the percentage of correct query-target pairs (true positives) as a function of the percentage of incorrect pairs (false positives) as we traverse the list of query-target pairs sorted by TOMTOM $p$-value or any of the other three method of combining columnwise scores. The solid and dashed lines correspond to width-normalized scores scores ($p$-values, arithmetic mean, and geometric mean), while the green dotted line represents sum of column scores. Each panel contains a plot computed using a different column similarity function at a sampling rate of $S/8$. Similar results are obtained for sampling rates of $S/2$, $S/4$ and $S/16$ (data not shown).
