# Supplementary material for: Quantifying similarity between motifs
Source: Genome Biol. 2007 Feb 26;8(2):R24. doi: 10.1186/gb-2007-8-2-r24 (PMC1852410; doi:10.1186/gb-2007-8-2-r24)
Supplement: Additional data file 6 — E value based retrieval rate for two additional significance levels (E-value less than 0.05 or 0.001) [file gb-2007-8-2-r24-S6.pdf]

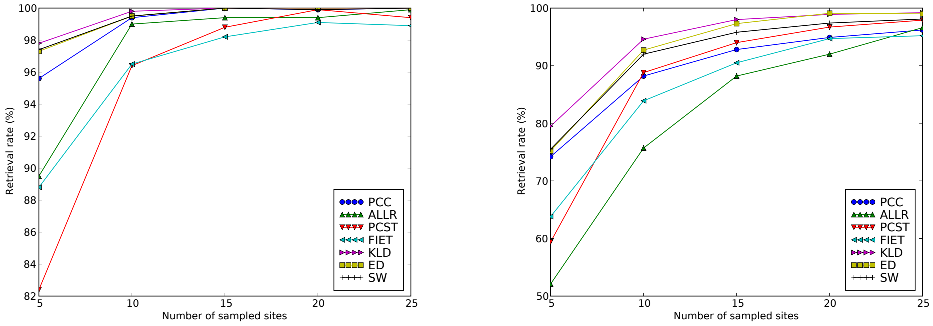

(A) E-value < 0.05 (B) E-value < 0.001

Figure 4: **E-value based retrieval rate.** The figure plots the percentage of query motifs that succesfully matched the correct JASPAR target as a function of the number of sites used to create the query motif. Here "success" means that the top-ranked motif is the correct target and has an E-value less than 0.05 (panel A) or 0.001 (panel B).
